# Effect of Climate on Photovoltaic Yield Prediction Using Machine Learning Models

**DOI:** 10.1002/gch2.202200166

**Published:** 2022-10-20

**Authors:** Alba Alcañiz, Anders V. Lindfors, Miro Zeman, Hesan Ziar, Olindo Isabella

**Affiliations:** ^1^ Photovoltaic Materials and Devices Group Delft University of Technology Mekelweg 4 Delft 2628 CD The Netherlands; ^2^ Finnish Meteorological Institute Meteorological Research Erik Palménin aukio 1 Helsinki 00560 Finland

**Keywords:** climate, forecasting, Köppen‐Geiger, machine learning, photovoltaics

## Abstract

Machine learning is arising as a major solution for the photovoltaic (PV) power prediction. Despite the abundant literature, the effect of climate on yield predictions using machine learning is unknown. This work aims to find climatic trends by predicting the power of 48 PV systems around the world, equally divided into four climates. An extensive data gathering process is performed and open‐data sources are prioritized. A website www.tudelft.nl/open-source-pv-power-databases has been created with all found open data sources for future research. Five machine learning algorithms and a baseline one have been trained for each PV system. Results show that the performance ranking of the algorithms is independent of climate. Systems in dry climates depict on average the lowest Normalized Root Mean Squared Error (NRMSE) of 47.6 %, while those in tropical present the highest of 60.2 %. In mild and continental climates the NRMSE is 51.6 % and 54.5 %, respectively. When using a model trained in one climate to predict the power of a system located in another climate, on average systems located in cold climates show a lower generalization error, with an additional NRMSE as low as 5.6 % depending on the climate of the test set. Robustness evaluations  were also conducted that increase the validity of the results.

## Graphical abstract

This work explores the effect of climate on photovoltaic (PV) yield prediction when using machine learning algorithms. A website with open‐source PV power data is created as result of the data gathering process. Forty eight PV systems worldwide divided into four climates are employed for this purpose. Systems in tropical and dry climates present the highest and lowest errors, respectively.

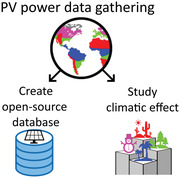

## 1 Introduction

Continuing with previous years’ trends, the global photovoltaic (PV) market grew again in recent years, despite the COVID‐19 pandemic.^[^ ^1^ ^]^ The total cumulative installed capacity recently passed the 1 TW threshold, entering the TeraWatt era of photovoltaics.^[^ ^2^ ^]^ This expansion can complicate the management of the electrical grid. PV is a variable energy resource due to its dependency on weather conditions. Rapid alterations between sunshine and clouds create fluctuations in the power generation, which result in voltage unbalance, voltage rise and voltage flickers in networks with high PV presence.^[^ ^3^ ^]^

Forecasting technologies can help grid operators with the scheduling and dispatching of this renewable energy source more effectively. Due to the stochastic nature of PV power generation, machine learning (ML) approaches have gained popularity in forecasting tasks.^[^ ^4^ ^]^ The main characteristic of ML algorithms is that the coefficients of the model are obtained automatically by using training data.^[^ ^5^ ^]^ There is a huge amount of algorithms inside this family, whose complexity ranges from linear regression techniques to deep learning neural networks.

Despite abundant literature, most studies consider <5 PV systems that are generally located in the same areas.^[^ ^6^ ^]^ Most researchers study systems in Europe, the US, Australia, or China, which are population‐dense areas, but receive relatively small solar intensity (except for Australia). Few studies have considered PV systems subjected to diverse meteorological conditions in order to study the effect that climate has on the performance of ML models.

Pasion et al. forecasted the PV power of 12 northern‐hemisphere locations subjected to seven different climate regions.^[^ ^7^ ^]^ The selected algorithm best predicted the data in a hot‐dry climate region, and a mixed‐humid climate region had the second‐best model performance, with coefficient of determination (*R* ^2^) scores of 0.968 and 0.962, respectively. In contrast, the model performance for the tropical rainforest site was the poorest with an *R* ^2^ score of 0.908. Do et al. forecasted two PV systems, one in Guadeloupe (North America, tropical climate) and the other in Lille (Europe, mild temperate climate).^[^ ^8^ ^]^ They discovered that the system in Lille required a longer training duration but achieved a lower normalized root mean squared error of 10.69 % compared to the one in Guadeloupe (error of 11.97 %). In ref. [9], two systems located in tropical (Singapore) and mild temperate (Australia) climates were also considered for a probabilistic forecasting model. As opposed to the previous study, they reported worse metrics for the system located in the mild temperate climate, due to the highly variable Australian weather and thunderstorms in the summer season. Zhang et al. considered three distant PV systems located in the USA, Denmark, and Italy.^[^ ^10^ ^]^ Because of the unique weather and climate characteristics of each location, the modeling parameters and best features of the optimum ML model differed between sites. Finally, in ref. [11] the forecasting performance of an American and a Chinese PV plant were compared. The authors reported that the station in the USA achieved a lower mean absolute percentage error of 6.65 % compared to the 9.31 % error in the Chinese PV power station. In most of these works, only a couple of systems per climate are considered, hence the reported climatic trends have a high dependency on the specifics of each site.

The objective of this work is to study the effect that climate has on machine learning predictive models. For this purpose, a data set of 48 PV systems spread around the world is considered. These systems are selected so that they are equally divided into four climates and as spread as possible. Several ML algorithms are trained on this data and the results are analyzed to identify climatic trends. The best algorithm is selected for further evaluations, which consist of quantifying the increase in error when the climate of the training and test set do not coincide, and of exploring how robust the results obtained are.

The rest of the paper is organized as follows. Section 2 details the data set employed in this work. It explains the process of data gathering, the considered inputs, data preparation, and data exploration. Section 3 describes the methods employed, which are mainly the ML algorithms, but also includes the hyperparameter tuning process and the metrics. The main results are analyzed in Section 4, where some evaluations are also conducted to show the generalization and robustness of the model, amongst other characteristics. Finally, main conclusions are presented in Section 5.

## 2 Data Set

This section explains the main characteristics of the data set employed. The first step consists of the data gathering process, where open‐source data sets were prioritized. Once the data is selected, their main characteristics are explained. The data is later cleaned and explored before running the ML models.

### 2.1 Data Gathering

Due to the nature of this work, a large and important part of this study was the process of data gathering. In order to find trends in climate and have enough statistical power, a data set of sufficiently high number of spread systems was needed, with the same amount of systems per climate. In this search, open‐source PV data was prioritized in order to increase the reproducibility of the results. The process of data gathering is explained in the supporting information. All the open data sources can be easily checked in a website www.tudelft.nl/open-source-pv-power-databases developed by the authors. Additionally, anyone interested in sharing their data is open to contribute to it. This could help tackling one of the gaps in the discipline: lack of comparison between studies.^[^ ^17^ ^]^ Given that ML algorithms are sensitive to many factors such as location and data resolution, it is generally unfair to make a comparison between studies. One solution to this issue would be to employ the same data and be as transparent as possible with the parameters and techniques employed. This database would allow researchers to improve results from previous studies, and to reduce the limitations of data.

### 2.2 Data Characteristics

**Figure 1:**
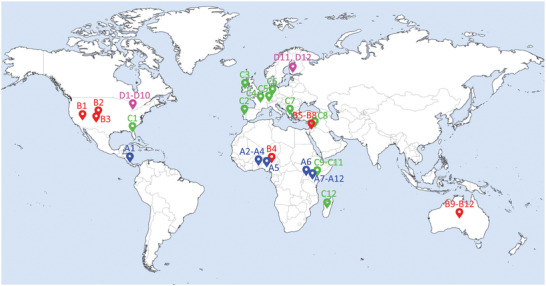
Location of the 48 PV systems employed in this study with their identifications. The colors represent the major KG climate that each system is subjected to.

After the data gathering process, a total of 48 PV systems as spread as possible were chosen, situated in 18 different countries. The location of each system can be seen in **Figure** **1**. To distinguish between meteorological conditions, the major types from the Köppen‐Geiger (KG) climate classification were employed:^[^ ^18^ ^]^ tropical (A, blue), dry (B, red), mild temperate (C, light green), and continental (D, magenta). The major type polar was left out of this study since no PV system data were found there. The capacity of each system is contained in **Table** **1**.

**Table 1:** Capacity of the 48 selected PV systems.

Capacity (kW_*p*_)	1	2	3	4	5	6	7	8	9	10	11	12
A	5.5	8.2	6.9	5^1^	6^1^	5^1^	4.9	1.5^1^	6.0	5.0	2^1^	9.8
B	6.0	2.7	6.0	9.8	80^1^	75^1^	190^1^	10^1^	5.0	5.0	226.8	22.6
C	6.0	7.0	6.0	4^1^	8.0	20.6	140^1^	7.8	6.0	2^1^	2^1^	4^1^
D	6.0	10.0	7.6	8.8	7.5	7.6	7.6	5.0	7.6	7.6	21.0	20.3

^1^Capacity is estimated from the PV power data

Regarding the origin of the data: A1 was provided by Dr. Victor Vega from the University of Costa Rica; all American systems (B1‐B3, C1, D1) were obtained from PVDAQ by the National Renewable Energy Laboratory;^[^ ^19^ ^]^ all Australian data (B9‐B12) were downloaded from the Solar Centre of Desert Knowledge Australia;^[^ ^20^ ^]^ the German system C6 can be found in Sunny Portal;^[^ ^21^ ^]^ and the Finnish systems are installed in the Finnish Meteorological Institute. The remaining data was provided by 3E.^[^ ^16^ ^]^

The arduous task of data gathering severely limited the accuracy of this work. Some of the gathered data included very few meteorological measurements and limited system information. For instance, 3E data only included global irradiance, ambient temperature and wind speed as measured variables. Therefore, despite some systems had more descriptive parameters available, these were not included in the study. All PV systems needed to have the same meteorological and system information, otherwise it could have affected the robustness of the results. Overall, it was decided to sacrifice some meteorological parameters in exchange for a higher number of systems that would assure statistical robustness to our climatic trends.

Model inputs consist of hourly resolution clear‐sky irradiance, previous hour measurements of global irradiance, temperature and wind speed, power produced during the three previous hours and power produced one day before. No system characteristics were included in the model since very limited data was known from some systems due to privacy issues. Moreover, since no more descriptive meteorological parameters were available for all systems, the PV power produced during previous instants of time was included as a feature to provide additional information to the algorithm. The decision of selecting the power produced during the three previous hours and one day before was taken after looking at the changes in performance (not shown here). Including three previous hours and one day before yielded to slightly lower errors than removing previous hours in most algorithms, although some started showing the negative effects of cross‐correlated features. The clear‐sky global irradiance was implemented using the pvlib library.^[^ ^18^ ^]^ This variable represents the geographical dependency of the sites and irradiance patterns in time in a more effective way than with features such as latitude, longitude, sun azimuth or sun altitude.

For most systems, the meteorological data was gathered on site and obtained from the same source as the PV power. For the German open‐source PV system, there was no onsite weather data accessible, so an inverse interpolation method was applied to nearby meteorological stations provided by the German Weather Service.^[^ ^22^ ^]^

### 2.3 Data Preparation

Once the data was gathered, it had to be prepared for the ML algorithms. Several cleaning steps were therefore performed, consisting of:

- Units standardization of all measured data.
- Making sure that variables were within limits: positive PV power, no irradiance at night, ambient temperatures <100 °C etc.
- Removing days with no measurements.
- In presence of missing values, if there were three or more consecutive absent measurements, they were removed, while sporadic missing points were interpolated.
- Removing outliers. For ambient temperature and wind speed, interquartile range (IQR) was employed. A different IQR constant (usually set to 1.5) was found experimentally for each feature. This measure could not be employed for PV power and irradiance due to their low average. For those variables, values that were 25 % higher than the 99 % quantile were considered outliers.

Each system's data was individually explored (not shown) in order to detect any abnormal behaviour and check that the data cleaning process was properly implemented. Since the data availability was different for each system, after the cleaning process it was ensured that all systems had at least 75 % of a year of data, and a maximum of two years of data.

The next step consisted of data normalization, an essential requirement for some ML algorithms like Support Vector Regression that require a similar range for all features. All inputs were scaled between zero and one considering the minimum and maximum of each series. Finally, the data for each system was randomly split into 70 % of training set and 30 % of test set.

### 2.4 Data Exploration

Before diving into the methodology, it was also interesting to try to find possible differences in behavior between climates. For that reason, the correlation of PV power with each of the model inputs for each PV system was computed. Correlation is a value between

**Figure 2:**
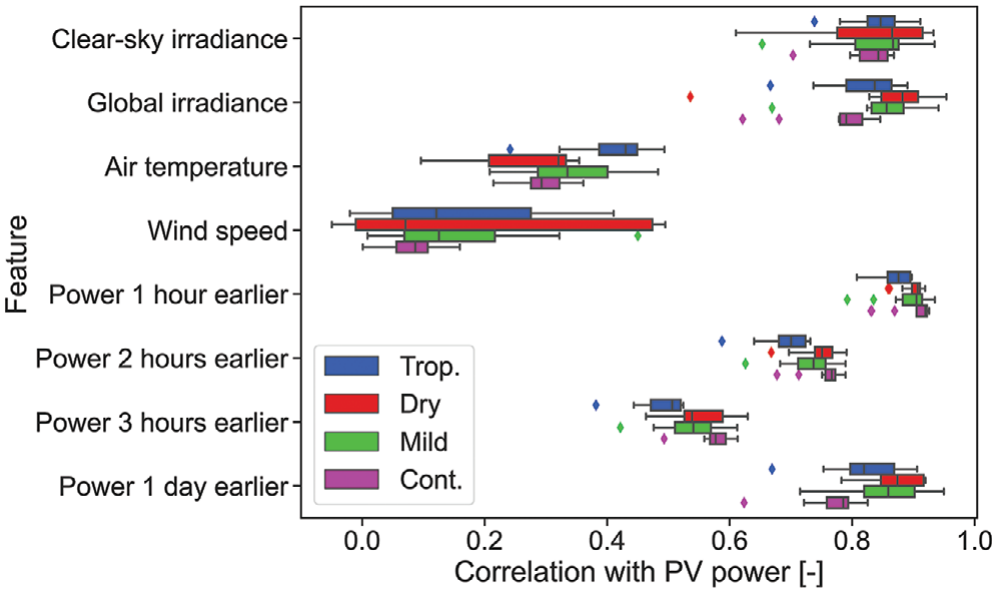
Correlation of PV power with each input feature for all 48 systems. The systems have been colored depending on the major KG climate that they are subjected to.

‐1 and 1, and indicates the similarity between two vectors. The correlation results were grouped based on climate in order to find meaningful trends, as can be seen in **Figure** **2**.

Irradiance is highly correlated with PV power, showing medians >0.84 and 0.79 for clear‐sky and global irradiance, respectively. In the case of systems located in dry climates, these two variables are more highly correlated than for the rest:

0.87 on average for both variables as compared to 0.86 for mild, 0.84 for tropical, and 0.82 for continental systems. Since clear‐sky irradiance is computed analytically, there is no time shift with respect to PV power. This makes it a highly explainable feature of the PV power in clear‐sky days.

The other two measured variables, air temperature and wind speed, have lower importance for the ML algorithms. In fact, wind speed has on average a correlation of 0.14 with PV power, suggesting no importance for most systems. Seven systems even show a negative correlation, five of which are in a dry climate, but it is so low (minimum of −0.05) that it indicates no relationship between variables. Regarding air temperature, one can observe a high dependency with climate, since the median correlation for tropical systems is of 0.43, while in the remaining climates it drops to

0.30.

The hourly previous instances of PV power show an expected correlation, dropping as the distance in time increases. Tropical systems show the lowest correlation of all climates, with a value of

0.87 while it increases to 0.9 for the other climates. This indicates that the intraday weather variability in tropical areas is very high compared to other climates since the PV power can be very different between hours. Looking now at the correlation with power one day earlier, tropical climates also depict a low correlation (0.82), but continental climates obtain an even lower one (0.78). Following the same reasoning as before, systems in continental climates show a high daily weather variability.

## 3 Methodology

This section explains the algorithms employed for predicting the PV power, and how their parameters were optimized (hyperparameter tuning). The metrics employed for model assessment are also defined.

### 3.1 Algorithms

This subsection briefly explains the working principle of the algorithms employed to predict PV power in this work. A more in depth and mathematical explanation is out of the scope of this publication.

Persistence algorithms assumes that nothing changes between the current time step and the following.^[^ ^23^ ^]^ Equation 1 describes the model, where *P*(*t*) is the PV power produced at time step *t*. This simple algorithm can achieve low errors when forecasting PV power in the very short‐term.^[^ ^24^ ^]^

(1) P(t)=P(t−1)

The second algorithm is the high‐order polynomial regression model. The relation between the independent variable (*P*(*t*) in our case) and the features is modeled by a polynomial of degree *d*. This statistical model can be extended to machine learning by determining the parameters through the training data set.

One issue of regression algorithms is that they tend to overfit the data. The model focuses too much on the training set and it is unable to generalize to unseen data. To solve this issue, regularization techniques were developed which keep the parameters low. For instance, ElasticNet penalizes the parameters through the 1‐norm (absolute value) and 2‐norm (root squared). The user can choose the ratio between these two penalization terms *l*_*r*_ as well as the amount of penalization α.

Support Vector Machine (SVM) is an algorithm originally developed to solve classification problems, and extended for regression under the name of Support Vector Regression (SVR). The objective of SVM in classification problems is to create a boundary between groups with the highest possible margin. SVR follows the same principle of maximizing the margin: it fits a hyperplane to the data with a margin of tolerance *ϵ*.^[^ ^5^ ^]^ Usually, a hyperplane does not properly describe the data. In such cases, two simultaneous approaches are taken. First, a penalization term C is introduced to the samples which are outside the hyperplane, so that the minimization function has a motivation to fit all points inside the hyperplane. The second approach is to map the data to another feature space, where it may be easier to fit a hyperplane. This is done via a kernel, a function that helps SVR to solve non‐linear problems. The most common kernel functions are polynomial, radial basis function, and sigmoid, expressed together with its parameters in **Table** **2**. In these equations, *x* is the real output while *x*′ is the predicted one.

**Table 2:** Possible kernels for SVR

Kernel	Expression	Parameters
Polynomial	(γ · 〈*x*, *x*′〉 + *r*)^*d*^	γ, *r*, *d*
Radial basis function	exp (−γ · ||*x* −*x*′||^2^)	γ
Sigmoid	*tanh*(γ · 〈*x*, *x*′〉 +*r*)	γ, *r*

Random forest is a combination of decision trees. A decision tree is an algorithm that recursively partitions the data space depending on its value, and a simple prediction model is then fitted within each partition.^[^ ^26^ ^]^ To avoid overfitting, a random forest fits a number of decision trees *n*_*estimators*_ on various subsets of the dataset and then averages them. *max*_*samples*_ controls the size of the subset employed to build each tree. Several characteristics of the built trees can be controlled as well, such as the maximum number of divisions (depth) of each tree *max*_*depth*_, the amount of features considered for each split *max*_*features*_, the minimum number of samples required to be at a leaf (final) node *min*_*samples*, *leaf*_ and the minimum number of samples required to make another division *min*_*samples*,*split*_.

The last applied algorithm is Extreme Gradient Boosting, or XGBoost. It also combines several decision trees, but contrary to random forest, XGBoost combines the trees on the go instead of at the end.^[^ ^27^ ^]^ The first decision tree estimates an output that resembles as much as possible the real one, based only on the input dataset. In the next iterations, new decision trees are built by giving higher attention to large‐error predictions. There are several parameters to optimize in this algorithm although we focused only on two: *colsample*_*bytree*_ and α. The first parameter adds randomness to the model, using a similar strategy as that of random forest, and determines the fraction of randomly selected inputs employed to train each tree. α is a 1‐norm regularization term, as in ElasticNet.

All these algorithms have been implemented in python using the libraries *sklearn* ^[^ ^25^ ^]^ and *xgboost*.^[^ ^27^ ^]^

### 3.2 Hyperparameter Tuning

In order to improve the predictions, a hyperparameter tuning process for each algorithm and PV system has been performed. This process consists of finding the optimum set of parameters for each site. The parameters considered for each algorithm are the ones mentioned in the previous section. The range of each tuned hyperparameter can be found in the Supporting information. Each model was tuned using five‐fold cross‐validation with randomized search.

The results of the hyperparameter tuning have been analyzed in order to identify climatic trends. However, no clear relation between climate and optimum hyperparameter has been found.

### 3.3 Metrics

This section describes the metrics employed to evaluate the model performance. Several metrics are needed to understand the origin of the prediction errors. Since the PV systems have different rated capacities, all metrics employed are normalized so results can be compared between sites.

The error is computed as the difference between real values *y* and predicted ones $\hat{y}$. This operation results in a time series, which is hard to compare and interpret. Several operations have been performed to interpret this time series as a single value. The mean absolute error (MAE) takes the average of the absolute difference of time series to inform how off the predictions are on average. The root mean squared error (RMSE) goes a step further by taking the square of the difference to signal large errors in the prediction. The mean bias error (MBE) operates differently by adding the standard difference and indicates whether the predictions are under‐ or overestimating. These metrics can be normalized by considering the average of the actual values $\bar{y}$, yielding the normalized mean absolute error (NMAE, Equation 4), normalized root mean squared error (NRMSE, Equation 5) and normalized mean bias error (NMBE, Equation 6). In these equations, *n* corresponds to the number of samples.

(2) $$NMAE=\frac{1}{\bar{y}\;\cdot \;n}\;\sum_{i=1}^{n}|y_{i}-{\hat{y}}_{i}|$$

(3) $$NRMSE=\frac{1}{\bar{y}}\;\sqrt{\frac{1}{n}\sum_{i=1}^{n}{\left(y_{i}-{\hat{y}}_{i}\right)}^{2}}$$

(4) $$NMBE=\;\frac{1}{\bar{y}\;\cdot \;n}\overset{n}{\underset{i=1}{\;\sum }}(y_{i}-{\hat{y}}_{i})$$

*R* ^2^ score or coefficient of determination measures the goodness of a fit of a model. The best value it can take is 1, hence the higher the score, the better. Its expression can be found in Equation 7.

(5) $$R^{2}=1-\frac{\sum_{i}{\left(y_{i}-\overset{}{{\hat{y}}_{i}}\right)}^{2}}{\sum_{i}{\left(y_{i}-\bar{y}\right)}^{2}}$$

All metrics have been multiplied by 100 % so they are expressed in percentage terms.

## 4 Results

This section presents the results found in this work. The first subsection explains the main results obtained, focusing on finding climatic trends. Subsection 4.2 explores how well a trained model can predict the PV power of a different system. The robustness of these results is explored in Subsection 4.3, to increase the credibility of the obtained results. Finally, the last subsection sorts the obtained results based on a more suitable climate classification: the Köppen‐Geiger‐Photovoltaic climate classification.^[^ ^28^ ^]^

### 4.1 Climatic Trends

**Figure 3:**
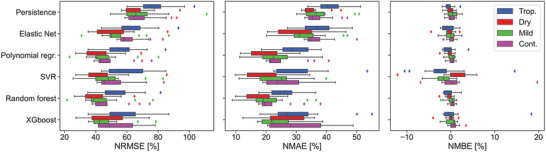
Performance of the ML models (and persistence) in terms of NRMSE, NMAE, and NMBE for all 48 PV sites. Each color represents a KG climate.

Once all algorithms were trained and optimized for each of the 48 PV systems, their performance was evaluated in the test set. The results for each model in terms of NRMSE, NMAE, and NMBE have been reported in **Figure** **3**, color‐coded based on climate.

Looking first at the models’ performance, their ranking is independent of climate and of metric. Persistence shows the highest NRMSE of 70.4 % on average, followed by ElasticNet (57.6 %) and SVR (51.6 %), while polynomial regression, XGBoost, and random forest show similar performance. The latter is selected as the best algorithm with a lower average NRMSE of 46.2 % compared to 48.4 % and 47.4 % for polynomial regression and XGBoost, respectively. Similar conclusions can be reached by looking at the NMAE. The fact that polynomial regression is better than ElasticNet, despite the latter being an improvement of the former, hints that the amount of data is high enough so that there is no overfitting. This was already elucidated by the hyperparameter tuning results (not shown), where the parameter α of ElasticNet, which indicates regularization, was close to zero for all sites.

In terms of NMBE, SVR depicts the worst results of all. This low performance can be due to the cross‐correlation of input features. Regarding climatic trends, tropical and mild systems are more prone to being underestimated (median NMBE for all models of

0.21 % and

0.42 %, respectively) as opposed to those in continental and dry climates (median NMBE of 0.38 % and 0.35 %, respectively).

Despite depicting the highest NRMSE, persistence can provide climatic information. By definition, low persistence errors are obtained when the atmospheric conditions remain unchanged. Climates that show on average low persistence errors indicate low hourly variability in PV power. Continental systems depict the lowest median NRMSE of 62.5 % when using persistence, which was already expected considering that the systems in this climate also have a high correlation of 0.92 with the previous PV power value, Figure 2. Dry and mild systems also show a low median NRMSE of 65.0 %, again due to the 0.91 correlation of PV power with its previous value. This indicates that the changes in PV power from hour to hour are low. On the other hand, systems located in tropical areas show a high weather variability between hours, hence their high median NRMSE of 74.5 % and low correlation of 0.87. For instance, in Costa Rica the hourly and monthly rainfall patterns can be very fluctuating, without a defined pattern during the diurnal period.^[^ ^29^ ^]^

Looking now at climatic trends, on average systems located in dry areas are the ones that report the lowest NRMSE (47.6 % for all models), while those located in tropical areas show the highest NRMSE (60.2 %). Although both climates are characterised by high ambient temperatures and high irradiation, the higher humidity and precipitation of tropical areas negatively affect not only the performance of the PV systems but also their prediction.^[^ ^28^ ^]^ Systems in mild and continental climates show similar average NRMSE of 51.6 % and 54.5 %, respectively.

These results could be explained by a lack of climate‐specific features in the model. In particular, humidity, rainfall, snowfall, and dust. Unfortunately, this section could not be verified due insufficient data, but we can rely on literature for this hypothesis.

By definition, dry climates are characterised by a lack of available water.^[^ ^30^ ^]^ Variables such as humidity, rainfall, and snowfall will therefore have a low impact in the prediction of PV power. On the other hand, dust was selected as a relevant feature for the power prediction of a system located in the dry State of Qatar.^[^ ^31^ ^]^ Correlation of PV power with ambient humidity for that system was 0.24, while with cumulative dust was of

0.56. It is likely that the addition of dust in the ML models would decrease the prediction error.

For tropical PV systems, not only dust but also humidity and rainfall affect the production (and hence the prediction) of PV power. Considering the location of the selected PV systems, and that the highest dust accumulation occurs in the Middle East and North Africa,^[^ ^32^ ^]^ the selected dry systems are as affected by dust as the tropical ones. Considering the dust accumulation around the globe, the addition of dust as a variable would decrease the error for systems in tropical and dry climates, while it would barely have any impact in mild and continental systems. The addition of humidity would also have a positive impact in the prediction accuracy for tropical systems. In ref. [33] the correlation of ambient humidity with PV power for a tropical system was of

0.43, which according to Figure 2 is higher than the median correlation (in absolute value) with wind speed of 0.12. Moreover, tropical places usually show seasonal weather variations substantially different from other climates.^[^ ^7^ ^]^ Finally, by definition tropical climates have significant precipitation.^[^ ^30^ ^]^ Overall, the lack of humidity, rainfall, and dust as variables in the ML models could explain the lower errors achieved for tropical systems.

Regarding the two remaining climates, they are only distinguished from one another by temperature.^[^ ^30^ ^]^ The addition of humidity and precipitation would have a positive effect in the prediction for both climates, although the magnitude of this effect would differ depending on the location of each system. In ref. [7], the variable importance ranking for several systems depended on their location even when they were subjected to the same mild or continental climate. For one mild and one continental system, humidity was ranked as more important than temperature. The variable that could explain the lower prediction error of mild systems with respect to continental ones is snowfall. By definition, snowfall is more common in continental climates than in mild ones. Awad et al. found that *R* ^2^ increased from 0.93 to 0.96 when including rain and snow in their predictive model for Canadian systems.^[^ ^34^ ^]^ They claimed that snowfall helped the ML model learn the differences in fluctuating conditions between the snow and non‐snow seasons. Similarly, Böök et al. reported that, for the two Finnish systems included in this study, the average *R* ^2^ of their prediction during snow‐free days was of 0.995 while it dropped to 0.640 during snow cover periods, which represent roughly 13 % of the data points.^[^ ^35^ ^]^

As a final reflection in this subsection, the errors found are quite high. Even when considering the best algorithm, random forest achieves a median 41.8% NRMSE and a median 19.7% NMAE. Comparing with literature, Pujić et al. achieved an NMAE of 8.6% when using random forest.^[^ ^36^ ^]^ Ferlito et al. achieved an RMSE with random forest ranging between 46.5 W and roughly 82.0 W, depending on the year, for a 1 kWp PV plant.^[^ ^37^ ^]^ In order to compare fairly these results, we select a representative PV system with known capacity. We choose system B3 with an NRMSE of 41.0%, close to the median. The RMSE for B3 is 0.57 kW, and has a capacity of 6 kWp. Therefore, the representative RMSE is 94.8 W/kWp, while for Ferlito et al., this value was of 46.5‐82.0 W/kWp. There may be several reasons for this underperformance. First, the amount of information employed, as already suggested. Here we relied on only a couple of general meteorological features and no system information. The models were not tailored for each PV system, beyond the hyperparameter tuning and a generic cleaning process. A more powerful ML algorithm, such as an Artificial Neural Network, may also reduce the error. For instance, in the average RMSE was reduced from 167 to 164 when using Long‐Short Term Memory instead of random forest.^[^ ^38^ ^]^ However, the objective of this study lies on the comparison between climates rather than on obtaining the lowest possible error per system.

### 4.2 Flexibility to Other Climates

The objective of this subsection is to find how flexible the machine learning models are against climate. We will find how the error increases when an ML algorithm trained with data from a system located in a certain climate predicts the power of a system located in a different climate. The final goal is to quantify the increase in error when an ML model is applied to unseen systems in different climates, in order to observe hidden relations and similarities. By no means, it is our objective to substitute this general model by the individual ones.

For this study, five new data sets were created, and an ML algorithm was trained with each one. The first data set includes data from all 48 PV systems, and the resulting model is called *Universal* model, since it should be valid for all systems. Then we construct one data set per climate, which includes the data from all 12 systems in that climate. For each data set, the data was randomized and the amount of points was restricted to 5 years due to computational limitations.

**Figure 4:**
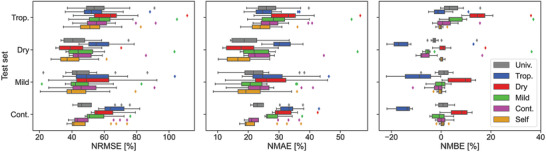
Performance of the universal, climatic and own models for all 48 PV systems.

The selected algorithm for this study is random forest, given its results in the previous subsection. Random forest parameters were first optimized for each set and then the algorithm was trained with each of the five new data sets. The PV power was then predicted for all 48 PV systems using the five newly trained models. **Figure** **4** shows the results for all models. In this figure, the colors and legend indicate the model used, while the vertical axis indicates to which climate the test system belongs to. The performance of random forest when trained with the same PV system (the model used in the previous subsection) has also been added to the graph for comparison purposes with the label of Self.

As expected, when the training and the test sets coincide (Self label, yellow color), the performance is optimum (average NRMSE of 46.2 %). The second best performance is obtained when the climate of the test and training sets coincide, which results in an average NRMSE of 47.9 %. The third best option is the universal model (grey color, NRMSE of 49.8 %), which includes data from all PV systems.

Up to this point, all results are expected. The performance decreases as the similarity degree of the training set with the test set decreases. We define similarity degree as the percentage of data belonging to the target system that is included in the training set. In the Self case, the similarity degree is the maximum, 100 %, since the system of the test and training set is the same. When the climate of the test and training sets coincide, the similarity is

8.3 % (one over twelve). In the case of the universal model, a fourth of the PV systems are from the same climate as that of the test set, thus the similarity is decreased by a factor of four, to 2.1 %. For the remaining conditions, the similarity degree is 0 %.

Therefore, the real flexibility experiment starts when testing a model trained with data in one climate on a PV system located in a different climate. From the graph, one can see that the continental model is the most flexible of all. This is seen for instance in the case of the dry test set, the continental model obtains a mean NRMSE of 48.8 %, lower than the mild or tropical models (49.9 % and 56.8 %, respectively). This higher flexibility of the continental model could be explained because it relies strongly on the PV power produced during the previous hour and because continental systems are subjected to a diverse but stable type of days. With similar reasoning, the mild temperate model also achieves good results. In the case of the dry model, since precipitation rarely occurs, it is not as accurate as continental or mild models during rainy days. Lastly, the tropical climatic model is the one that generalizes worst due to the challenging conditions in which it has been trained.

One could argue that this experiment is of no use, since it was already expected that if the training and test sets do not coincide, the accuracy will be lower. However, the main interest is the quantification of this lower accuracy and guidelines for the choice of an alternative training set in case the target PV system does not hold enough training data. Alternative approaches could be employed, such as developing a physical model or using persistence during the first months of operation, however here we aim to quantify the consequences of using a trained ML algorithm on a different test set.Assume that we want to predict the PV power of a recently installed system located in a tropical climate using an ML model. There is not enough data yet to train an ML algorithm and develop a model for that system. The only available data consists of two PV systems located in dry and mild temperate climates. From this subsection's discussions, lower errors are expected if a model trained with the mild system data is employed. That is only during the first months of operation, until sufficient data is gathered from the original PV system to create a model of its own. Following this example, we could estimate the increase in error made when using one system or the other. In terms of NRMSE, the additional error made when training with the mild system would be

6.5 % while in the case of the dry system, the additional error would increase to 10.0 %. This increase in NRMSE for all possible combinations are shown in **Table** **3**.

**Table 3:** Increase in NRMSE [%] in each of the combinations with respect to the performance of the *Self* case.

System's KG	Own climate	Univ.	Trop.	Dry	Mild	Cont.
Tropical	1.4	3.5	–	10.0	6.5	5.6
Dry	2.3	4.3	16.5	–	9.3	9.1
Mild	2.3	3.5	9.8	8.9	–	5.8
Continental	1.5	3.5	17.7	12.1	9.3	–

Note that these numbers are averages, therefore the actual values in another situation can differ. However, they give an idea of the error increase that one can expect. Whether this increase in error is acceptable, depends on the user and especially on the alternative. This path offers a way for PV power prediction using ML techniques with only one day of system operation.

### 4.3 Robustness

Despite having tried to include a broad range of systems per climate, the results may not be robust enough. Some of the systems are located nearby, and unfortunately not the same amount of data points was available per system. In this subsection, therefore, we try to show the robustness of the reported results.

**Figure 5:**
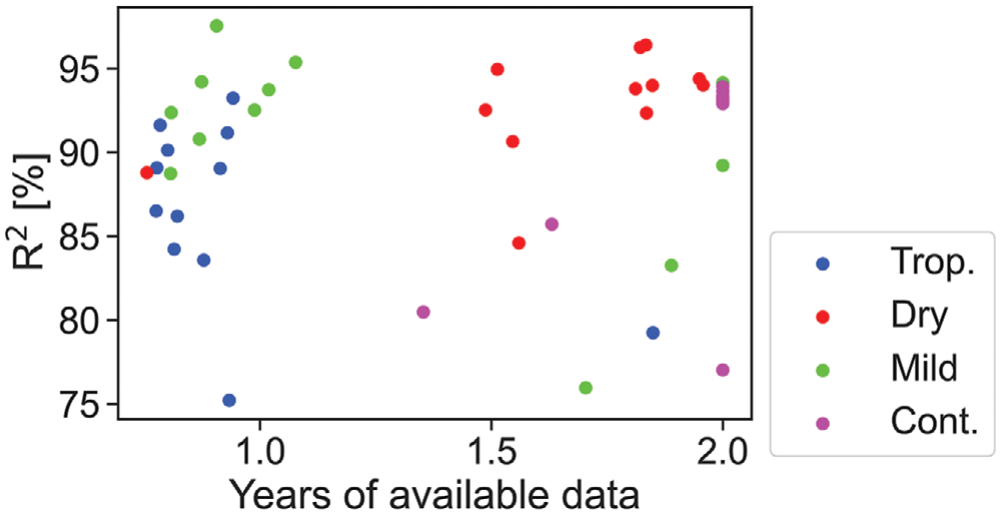
Coefficient of correlation as a function of number of years per system. The different colors represent the KG climate that the system is subjected to.

Twenty five percent of the systems had two years of data available after cleaning, while 37.5 % included less than a year. Since the amount of training data can affect the performance of the ML algorithms, we compared the amount of data points per system with their *R* ^2^. **Figure** **5** depicts the results for all 48 PV systems. One can observe that it is hard to find a meaningful relation between the amount of data points and the accuracy of the prediction, hence the different data availability of systems had no effect in this study.

In the flexibility experiment, Subsection 4.2, there is some contamination in the created data sets. Due to computational limitations, the system that is used as test set is included in the own climatic and universal training sets. That could under‐estimate the error made in the predictions. For that reason, more simulations were run showing how the difference in metrics is when including or excluding a certain system in the climate.

Let us select a random tropical PV system, such as A1. To test the robustness of the generalization study, two new data sets were created: the first included all tropical PV systems except for A1, while the second included the data from all PV systems except for A1. Random forest was trained using these two data sets, and the two models were used to predict the PV power of A1. Metrics were computed, and later compared with the metrics obtained for system A1 using the procedure explained in Subsection 4.2.

This procedure was repeated in two random systems per climate, adding up to a total of eight PV systems. When comparing the metrics of this procedure with those obtained from the generalization study, the results were best when the system is included, however, the differences are small. In terms of *R* ^2^, NMAE and NRMSE, the relative difference between the two conditions stays below 1.2 %, 2.5 %, and 3 %, respectively, for all systems. Therefore, despite existing some contamination in the created sets of the generalization study, the error is negligible.

The last detail to be looked at in this subsection is the amount of systems per climate. Despite all the data gathering efforts, 12 systems per climate might not be representative enough. To test this assumption, a new set of simulations were performed. They consisted of considering less systems per climate while checking whether the trends in performance were stable.

In the first iteration, instead of having 12 systems per climate, it is assumed that only 11 systems per climate were available. Twelve combinations per climate were therefore possible. The average NRMSE per climate of each of these combinations was computed. When comparing the NRMSE of 12 systems with all possible NRMSE of the 11 systems, one can identify if one of the systems is contaminating the results, or the trends are robust.

**Figure 6:**
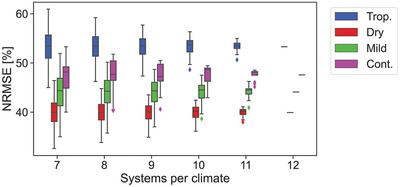
Evolution of the NRMSE as a function of the number of PV systems considered per climate.

This experiment was repeated considering down to seven systems per climate. **Figure** **6** shows the evolution in NRMSE as a function of the number of systems. Due to the possible combinations when the number of systems was lower than 12, the results are expressed with box plots. One can observe that in most combinations the trends reported in Subsection 4.1 are consistent: systems in tropical climates show the highest errors while those in dry ones depict the lowest ones. The error range in the box plots shows that increasing the number of systems makes the results more precise but has almost no effect in the ranking.

### 4.4 Köppen‐Geiger‐Photovoltaic Climate Classification

In this study, the major types of the KG classification have been used to divide the PV systems based on climate. This was chosen since it offers a simple classification into five categories, which was suitable for the problem at hand. However, recently Ascencio‐Vásquez et al. updated this classification particularly for PV, creating the Köppen‐Geiger‐Photovoltaic (KGPV) climate classification.^[^ ^28^ ^]^ This new classification includes another letter that indicates the strength of the irradiation level received: K‐Very High, H‐High, M‐Medium, and L‐Low. It was also based on an update of the KG classification which includes six major types, not five: A‐Tropical, B‐Desert, C‐Steppe, D‐Mild temperate, E‐Continental, and F‐Polar. Overall, this classification consists of 12 categories after merging combinations.

Considering the nature of this new classification, it would have been more suitable to employ these 12 categories. However, given the data gathering limitations, it would not have been possible to obtain enough number of systems per category to distinguish between trends.

**Figure 7:**
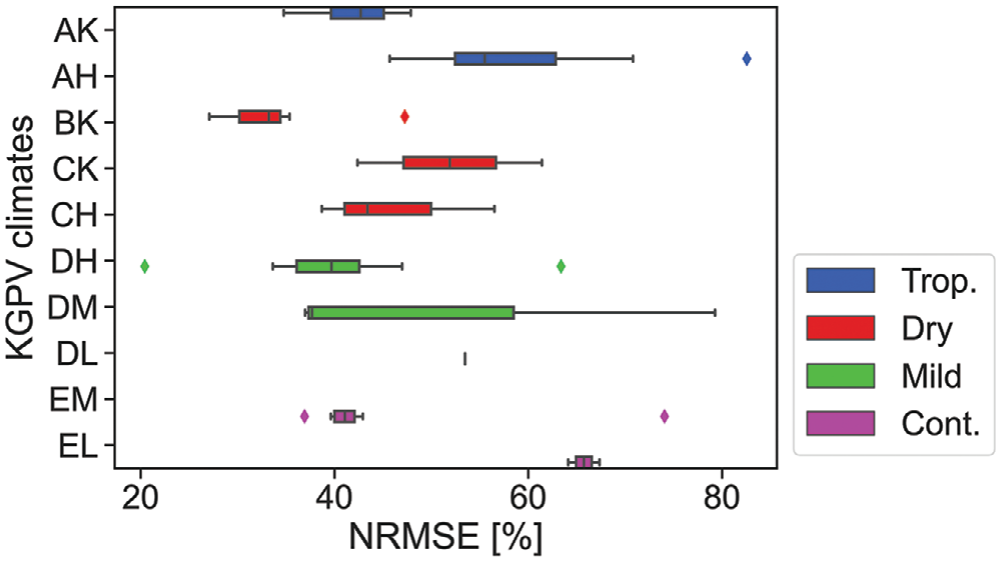
NRMSE as a function the Köppen‐Geiger photovoltaic climate classification. The colors represent the KG classification employed so far.

However, what can be done now, is to classify the results obtained in Figure 3 based on the KGPV classification. One has to be more critical with these new climatic trends, since it is unlikely that the systems are evenly distributed between these new 12 categories. It can still be the first step for possible future work. **Figure** **7** shows this classification in terms of NRMSE.

From the graph one can distinguish some subgroups for each climate. For continental climates one can see almost two perfect groups: EM and EL. The PV systems under the EL climate are more likely covered during longer periods by snow due to the lower irradiance. These are then harder to forecast since none of the input features explain the snow effects. One can also see how systems in the desert (BK) depict lower error than those in steppe areas (CK, CH). One can also observe that in general the higher the irradiance, the lower the error of the PV system. These could even override the previous trends, since from the graph, systems in DH climate show lower error than those in CH climate. This is opposed to the highest accuracy of dry climates found so far in previous sections.

Apart from the polar climate (F), which was excluded from this study, there is one KGPV climate missing: BH. This is logical considering that the dry climate has been divided into four categories, while the others have been divided into two or three. Climates DL, EL, CK are also highly unrepresented, which hinder any conclusions extracted in this work. It would be interesting to gather more data and extend this study with the KGPV climates.

## 5 Conclusion

In this work we have studied the effect that climate has on the performance of machine learning models for PV power prediction. We started by providing a database of open‐source PV systems that have been prioritized through this work, which has been published in a website form. Forty eight systems were selected, 12 per climate, and several machine learning algorithms were trained for each system. Results showed that random forest is the best algorithm of all tested, with an average NRMSE of 46.2 %. Climatic trends depicted how higher errors are achieved for tropical systems (NRMSE average of 60.2 % for several models), while lower ones are obtained for systems in dry climates (NRMSE of 47.6 %). We also studied the flexibility of these models, that is how the error increases when a model trained in a particular climate is used to predict the PV power of a system in another climate. Systems located in continental climates showed the lowest generalization error, which can be as low as an additional NRMSE of 5.6 %. Finally, several robustness experiments were performed to increase the confidence in the obtained results, and the recent Köppen‐Geiger‐Photovoltaic (KGPV) climate classification was used to further identify more climatic trends.

## Conflict of Interest

The authors declare no conflict of interest.

## Supporting information

Supporting Information

## Data Availability

The data that support the findings of this study are available from 3E. Restrictions apply to the availability of these data, which were used under license for this study. Data are available from the authors with the permission of 3E.
