# Supplementary material for: Effect of Climate on Photovoltaic Yield Prediction Using Machine Learning Models
Source: Glob Chall. 2022 Oct 20;7(1):2200166. doi: 10.1002/gch2.202200166 (PMC9818063; doi:10.1002/gch2.202200166)
Supplement: Supplementary file 1 — Supporting Information [file GCH2-7-2200166-s001.pdf]

Global Challenges
Open Access

Supporting Information

for *Global Challenges,* DOI: 10.1002/gch2.202200166

Effect of Climate on Photovoltaic Yield Prediction Using Machine Learning Models

Alba Alcañiz, Anders V. Lindfors, Miro Zeman, Hesan Ziar, and Olindo Isabella*

Supporting information
Effect of Climate on Photovoltaic Yield Prediction Using Machine Learning Models

Alba Alcañiz Anders Lindfors Miro Zeman Hesan Ziar Olindo Isabella*

1 Data gathering

In the following, a brief description of all found sources with individual PV system data is provided:

- PV Data Acquisition (PVDAQ), created by the National Renewable Energy Laboratory (NREL), provides access to photovoltaic performance data for systems throughout the US [1].
- The solar centre division of Desert Knowledge Australia (DKASC), a non-profit corporation of the Northern Australian territory, offers generated PV power of a wide range of PV technologies located in two different sites of the desert [2].
- Sunny Portal is a PV monitoring portal which allows PV system operators and installers worldwide to access system data at any time [3]. Despite the privacy of most of the systems, there is a set of example PV systems located in Germany which can be downloaded.
- The Global Energy Forecasting Competition (GEFCom) [4] conducted by Dr. Tao Hong considered the PV power forecasting problem in the 2014 edition. The employed data set is publicly available for researchers.
- Marion et al. made their data set of three American PV systems openly available. In their publication [5] one can find how to access the data.
- DuraMAT DataHub, also by NREL, is a collaborative framework where the public can provide and access PV data for durability studies [6]. Deep exploration of the website has not been carried out, but a data set of 9 bifacial tracking systems was found for download.
- The London Datastore open data-sharing portal [7] includes the Photovoltaic (PV) Solar Panel Energy Generation data set which contains voltage, current, power, energy and weather data from domestic sites with Solar Panels located in the city of London.
- Kaggle is an online machine learning community, subsidiary of Google, which allows users to find and publish data sets and to build models in a web-based data-science environment [8]. One can find several data sets related to PV power, which have been reported in www.tudelft.nl/open-source-pv-power-databases. For instance, *Horizontal Photovoltaic Power Output Data* data set was employed in an Energies publication [9]. It includes PV power and weather data for 12 Northern hemisphere sites over 14 months.
- The report published by the International Energy Agency regarding the Assessment of Performance Loss Rate (PLR) of PV Power Systems [10] required of PV system and weather data around the world. They made the dataset public in the OSF open platform which includes time series data from 18 PV systems located in different climates. For each plant, PV power, plane of array irradiance, ambient and module temperatures and sometimes wind speed data are available with time resolutions of 1, 10 or 15 minutes.
- Although generally not recommended since it is meant for software development and version control, GitHub is also employed to store data sets. For instance, the three individual systems in India with over 6 months of data and one minute resolution [11].

- The *Hathaway Solar Patriot House* is a project which monitors and models the performance of a sustainable house outside of Washington, D.C with a 6kWp photovoltaic system [12]. The collected data has been made available and includes a large number of measurements such as solar irradiance, DC electrical measurements from the PV array and battery, energy from and to the grid, energy consumption from electrical appliances, several temperatures and other measurements.
- The data provided by researchers Dr. Victor Vega from the University of Costa Rica (UCR) and Prof. Janez Krč from the University of Ljubljana can be directly downloaded from the developed website www.tudelft.nl/open-source-pv-power-databases.
- In order to access the two Finnish systems, Anders Lindfors from the Finnish Meteorological Institute (FMI) should be contacted.

For researchers interested in cumulative production instead of individual systems, there are also several choices:

- Belgian's electricity system operator, Elia, has had the initiative to provide open access to all of its public grid data [13]. Power generation data including generated PV power can be found for the whole country or by areas.
- Similarly, the German electricity market information platform SMARD provides actual generation over Germany, Austria and Luxembourg with 15 minutes resolution [14].
- Outside of Europe, the Electric Power Statistics Information System (in Korean) provides monthly data of power generation sorted by fuel type [15].
- Paul-Frederik Bach had the initiative of collecting data from several European system operators. One can find hourly time series of cumulative production in his blog [16].
- Also for Europe, Open Power System Data platform offers open data required by energy system models, including time series of solar power generation with up to 15 minutes resolution [17].

Other interesting sources, although not fully open-source, are PV CAMPER and pvoutput.org. PV CAMPER (Photovoltaic Collaborative to Advance Multi-climate Performance and Energy Research) [18] is a collaborative platform of PV institutions that share data within the community. There are entry requirements to access the community, but the platform was founded with the objective of data sharing and collaboration. pvoutput.org is a free service for sharing and comparing PV output data. Through the API one can retrieve generation data from any system after having shared data from an owned PV system and made a donation of minimum 15 AUD per year.

References

[1] N. D. Network, Pvdaq (pv data acquisition) api, URL `https://developer.nrel.gov/docs/solar/pvdaq-v3/`.

[2] D. K. Australia, DKA Solar Centre, URL `http://dkasolarcentre.com.au/`.

[3] S. S. T. AG, PV System List - Sunny Portal, URL `https://www.sunnyportal.com/Plants`.

[4] T. Hong, P. Pinson, S. Fan, H. Zareipour, A. Troccoli, R. J. Hyndman, *International Journal of Forecasting* **2016**, *32*, 3 896.

[5] B. Marion, A. Anderberg, C. Deline, J. Del Cueto, M. Muller, G. Perrin, J. Rodriguez, S. Rummel, T. J. Silverman, F. Vignola, R. Kessler, J. Peterson, S. Barkaszi, M. Jacobs, N. Riedel, L. Pratt, B. King, In *2014 IEEE 40th Photovoltaic Specialist Conference, PVSC 2014*. Institute of Electrical and Electronics Engineers Inc., ISBN 9781479943982, **2014** 1362–1366.

[6] NREL, Datasets - DuraMAT Data Hub, URL https://datahub.duramat.org/dataset.

[7] Photovoltaic (PV) Solar Panel Energy Generation data - London Datastore, URL https://data.london.gov.uk/dataset/photovoltaic--pv--solar-panel-energy-generation-data.

[8] Google, Kaggle: Your Machine Learning and Data Science Community, URL https://www.kaggle.com/.

[9] C. Pasion, T. Wagner, C. Koschnick, S. Schuldt, J. Williams, K. Hallinan, *Energies* **2020**, *13*, 10 2570.

[10] R. H. French, L. S. Bruckman, D. Moser, S. Lindig, M. van Iseghem, J. S. Stein, M. Richter, M. Herz, W. van Sark, F. Baumgartner, et al., Assessment of performance loss rate of pv power systems, **2021**.

[11] V. Mehra, Solar home system jharkhand india, **2016**, URL https://data.openei.org/submissions/681.

[12] P. Norton, E. Hancock, G. Barker, P. Reeves, Hathaway"solar patriot"house: A case study in efficiency and renewable energy, Technical report, National Renewable Energy Lab., Golden, CO (US), **2005**.

[13] E. Group, Elia: Belgian's Electricity System Operator, URL https://www.elia.be/.

[14] Bundesnetzagentur, SMARD- Market data, URL https://www.smard.de/en/.

[15] K. P. Exchange, Electric Power Statistics Information System (EPSIS), URL http://epsis.kpx.or.kr/epsisnew/selectMain.do?locale=eng.

[16] P.-F. Bach, , URL http://www.pfbach.dk/.

[17] N. N. Energieökonomik, T. U. of Berlin, E. Zürich, Open Power System Data, URL https://open-power-system-data.org/.

[18] S. Energy, Photovoltaic Collaborative to Advance Multi-climate Performance and Energy Research (PV CAMPER), URL https://energy.sandia.gov/programs/renewable-energy/photovoltaic-solar-energy/projects/photovoltaic-collaborative-to-advance-multi-climate-performance-and-energy-research-pv-ca
